# Histological comparison of demineralized bone matrix and the Ricinus communis polymer on bone regeneration

**DOI:** 10.1016/S1808-8694(15)31065-X

**Published:** 2015-10-22

**Authors:** José Rodrigues Laureano Filho, Bruno de Lira Castelo Branco, Emanuel Sávio Souza Andrade, José Ricardo de Albergaria Barbosa

**Affiliations:** 1 PhD. Professor of Bucco-maxillofacial Traumatology and Surgery - School of Dentistry - Pernambuco University - FOP/UPE. Mailing address: Prof. Jose Rodrigues Laureano Filho - Faculdade de Odontologia de Pernambuco - FOP/UPE Av. Gal. Newton Cavalcanti 1.650 Camaragibe PE 54753-220.; 2 DDS; Specialization Course Student in Bucco-maxillofacial Traumatology and Surgery - School of Dentistry - Pernambuco University - FOP/UPE.; 3 PhD. Professor of Oral Pathology - School of Dentistry - Pernambuco University - FOP/UPE.; 4 PhD. Professor of Bucco-maxillofacial Traumatology and Surgery - School of Dentistry -Piracicaba - University of Campinas - FOP/UNICAMP. School of Dentistry - Pernambuco University - FOP/UPE.

**Keywords:** calvaria bones, rabbits, ricinus communis, bone regeneration

## Summary

**A**im The aim of the present study is to make a histologic analysis the effects of a human demineralized bone matrix and a polyurethane resin derived from the Ricinus communis, on bone regeneration process. **Materials and Methods** For this, 24 rabbits were submitted to two surgical calvaria bone defects, one on the right and another on the left side of the parietal suture. The animals were divided in two groups. In group I the experimental defect (right side) was treated with a human demineralized bone matrix, while in group II the experimental cavity was treated with the polyurethane resin derived from Ricinus communis. The control defects were filled with the animal's own blood. The animals were slaughtered after subsequent periods of 04, 07 and 15 weeks. **Results** The histological analysis revealed that all groups (control and experiment), presented increased bone regeneration with time, but this repair was faster in the control group, even showing important decrease in defect thickness. **Conclusion** Both materials proved to be biologically compatible, however polyurethane was more slowly resorbed presented considerable better results when compared with demineralized bone matrix.

## INTRODUCTION

Bones are very hard conjunctive tissues; however, with great plasticity, bearing a highly complex metabolism, with repair potential involving ions, cells, hormones, proteins and nutritional factors^1^. Among the different processes of tissue repair, bone repair is one of the most complex, thus calling the attention of researchers in the field of Bucco-maxillofacial traumatology and surgery^2^.

Bone tissue bears a high capacity of spontaneous recovery when injured, and after a brief time span it is able to replace all the lost portion^3^. Nonetheless, in some cases of bone defects, sometimes extensive and unable to repair spontaneously, there is the need to use different surgical techniques in an attempt to correct the bone defect^4^.

Rebuilding these extensive lesions in hard tissues has always been a great challenge for science5. Different materials have been used for that, autogenous bone, freeze-dried bone, demineralized bone, as well as artificial or synthetic material^6^.

Demineralized and lyophilized bone grafts bring about fast healing of bone defects and have the property of inducing a considerable osseous neoformation, and do not have any antigenic property^7^. Demineralized and lyophilized bone grafts are disadvantageous in their shape and origin. Their particle size limit the amount of coronal apposition of this available bone in horizontal defects and any light tissue pressure may dislodge the grafted material8. One also has to consider the difficulty in obtaining viable human bone in large amounts, letting alone the fact that in many countries it is prohibit to trade human organs or tissue; it is of high cost and may transmit diseases ^3^.

In 1984, the Analytical Chemistry and Polymer Technology Group of the Engineering School of São Carlos developed a polyurethane resin of vegetal origin extracted from castor oil9. Polymers bear the advantage of being flexible in their processing and formulation, they bear excellent structural properties; they do not emit toxic vapors or irritants, and are biocompatible10. Henning et al. (1989)11 observed its biocalcification in vivo and in vitro. Carvalho et al. (1997)^12^ observed osteointegration of such polymer and reported the resorption and replacement of this polymer for bone tissue.

Studies state that the autogenous bone is the best graft material; however, many bone tissue replacements have been proposed in order to avoid donor site morbidity and the increase in operative time^14^, trying to create or take from nature those materials that bring about an increase in repair and bone neoformation, which are biocompatible and osseoinductors^15^. Therefore, the present study aims at histologically assessing the effects of a demineralized bone matrix of human origin and a polyurethane resin derived from castor oil on the process of bone repair

## MATERIALS AND METHODS

In order to do that, we used 24 albino New Zealand rabbits, with ages varying between 03 and 06 months, and average weight of 2.9kg.

These animals were divided in two equal groups (Group I and Group II), according to the type of material implanted: human demineralized bone matrix - DEMBO-NETM and polyurethane resin extracted from castor oil - AUG-EX®, respectively, in three observation periods. These materials were commercially acquired. DEMBO-NETM is a demineralized bone matrix produced by a bone bank (Pacific Coast Tissue Bank) located in Los Angeles, EUA, broadly commercialized in this country. The polymer extracted from castor oil, AUG-EX® is produced by Poliquil Araraquara Polímeros Químicos Ltda., Brazilian company that used the technological development achieved at the São Carlos Chemistry Institute of the São Paulo University to produce and trade this polymer with bone characteristics in Brazil.

The animals were kept in the animal handling facilities of the Dentistry School of Piracicaba - Unicamp. Since rabbits behave well in community, 4 to 5 animals were kept in each cage. Bed changes and the cleaning of cages, water dishes and food dishes were carried out daily, according to the protocol of cleaning and disinfection adopted by the Piracicaba Dentistry School - Unicamp. The animals were fed solid pelletized commercial rations, with high protein value (20 to 27%), and water ad libitum.

**Figure 1:**
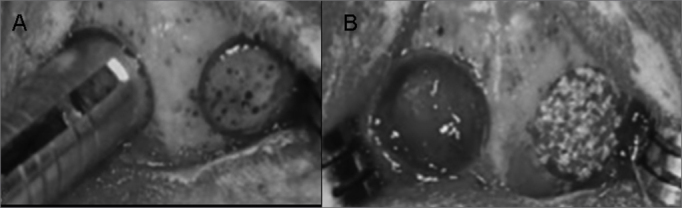
Experimental Surgery in the Rabbits' Cranial Vaults - Cavity perforation with trephine. B -Cavity filled with blood on the left side and with tested material on the right side.

Both groups had 12 animals equally distributed in three observation periods (4, 7 and 15 weeks). In each animal, two cavities were created in their cranial vault. In Group I, one of the cavities was filled with human-origin demineralized bone matrix (right side - experimental) and the other was filled with blood (left side - control). In Group II, one of the cavities was filled with polyurethane resin extracted from castor oil (right side - experimental) and the other one by blood (left side - control) (Figures 1A and 1B) (Table 1).

**Table 1:** Sample distribution on Group I (human origin demineralized bone matrix) and Group II (castor oil extracted polyurethane resin).

Observation Period	Group 1	Group 2
4 weeks	4 animals	4 animals
7 weeks	4 animals	4 animals
15 weeks	4 animals	4 animals

The animals were slaughtered at ^4^, ^7^, and 15 week intervals after surgery, in a total of four animals per group per slaughter period, as depicted on Table 2. Afterwards, the bone blocks were removed in order to prepare the slides from the bone cavities filled and perform the microscopic analysis, by means of Hematoxylin Eosin dye (H/E).

**Table 2:** Sample distribution according to the period.

Group	Slaughter
G1	4 weeks of post-op
G2	4 weeks of post-op
G1	7 weeks of post-op
G2	7 weeks of post-op
G1	15 weeks of post-op
G2	15 weeks of post-op

After dying and assembling the slides, they derwent light microscopy study, aiming at observing the repair that happened to the rabbits' skulls. The slides were then seen by a pathologist, separately, at the Dentistry School of Pernambuco (FOP/UPE). Pre-established parameters were then written down and the microscopic findings were then qualitatively analyzed. At this stage, the pathologist was unable to tell to which group the slides belonged to. This was possible because the slides were numbered in such a way that it did not identify the group, or the time at which the rabbit was slaughtered.

This study was referred to the Ethics Committee in Animal Experimentation of the Biology Institute at the University of Campinas - CEEA-IB/Unicamp, and was approved under protocol number 183-1, before the beginning of this project.

## RESULTS

### Histology Analysis

#### Four weeks - Control Group (demineralized bone)

**Figure 2:**
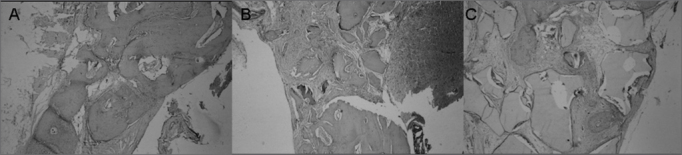
Slaughter period - 04 weeks (H/E - 100X) - A - Control Group B - Experimental Group: Dembone C - Experimental Group: Castor oil Polymer.

We mainly observed partial closure of the bone defect with an overlapping of fibrous connective tissue, osteoid and osteoblasts. In one of the individuals we also noticed an intense chronic inflammation (Figure 2A).

#### Four weeks - Experimental Group (demineralized bone)

Partial closure of the bone defect, with a large amount of fibrous connective tissue and bone neoformation on the margins (Figure 2B).

#### Four weeks - Control Group (castor oil polymer)

Fibrous connective tissue overlapping, with osteoid, osteoblasts, osteocytes, mature tissue and bone neoformation.

#### Four weeks - Experimental Group (castor oil polymer)

Fibrous connective tissue with mature bone tissue formation, osteoid and osteoblastic layer around the filling material (Figure 2C).

#### Seven weeks - Control Group (demineralized bone)

Fibrous connective tissue present with a large amount of osteoblasts intertwined among mature bone trabeculae and osteoid formation. In this group we assessed three individuals because during the microscopic evaluation we found the overlapping of intracranial tissue inside the bone defect.

#### Seven weeks - Control Group (castor oil polymer)

**Figure 3:**
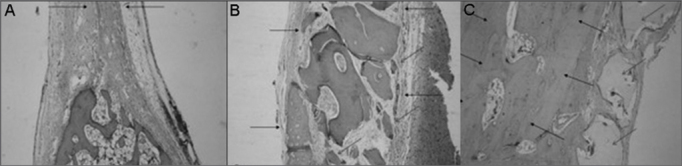
Slaughter period - 07 weeks - A - Control Group(H/E - 40X) B - Experimental Group: Dembone (H/E - 100X) C - Experimental Group: Castor oil Polymer(H/E - 100X).

Repair of a large part of the bony defect by mature bone tissue, observing the presence of fibrous connective tissue, large amount of osteoblasts and one visible reduction in bone thickness (Figure 3A).

#### Seven weeks - Experimental Group (demineralized bone)

Partial repair of bone defect by a large amount of fibrous connective tissue, there also was bone neoformation starting on the defect borders (Figure 3B).

#### Seven weeks - Experimental Group (castor oil polymer)

Almost total repair of the defect by mature bone tissue, with partial recovery of the internal cortical bone. Osteoblastic layering, osteoid and bone neoformation around the implanted material (Figure 3C).

#### Fifteen weeks - Control Group (demineralized bone)

**Figure 4:**
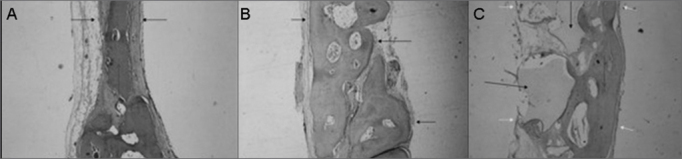
Slaughter period - 15 weeks (H/E - 40X) - A - Control Group B - Experimental Group: Dembone C - Experimental Group: Castor oil Polymer.

Defect repair by mature bone tissue with reduction in the thickness between the cortical layers and mild overlapping of fibrous connective tissue. Osteoid and large quantity of osteoblasts. In this group we analyzed three individuals because there was a problem during the technical preparation of the slides and one of the specimens was lost (Figure 4A).

#### Fifteen weeks - Experimental Group (demineralized bone)

Bone defect repair with mature bony tissue and fibrous tissue overlapping with reduction in the thickness between cortical layers. Numerous osteoblasts and intense osteoid depositing on the defect margins (Figure 4B).

#### Fifteen weeks - Control Group (castor oil polymer)

Complete defect repair by mature bone tissue, with reduction in the thickness of bone cortical layers. In this group we analyzed three individuals due to technical problems in making the slides; one of the specimens was lost (Figure 4C).

#### Fifteen weeks - Experimental Group (castor oil polymer)

Partial bone defect repair with mature bone tissue forming, fibrous connective tissue, and bone neoformation in the area adjacent to the implanted material; and in one of the implants we observed osteoclastic activity around the material.

## DISCUSSION

The study of material capable of replacing bone tissue has increased in health care professions^16^, especially in dentistry, where bone-integrated implant treatments and a greater awareness about the importance of bone reconstruction in patients who undergo mutilating procedures has increased recently^17^.

Autogenous bone graft is still considered the best means to replace bone loss above the necessary critical limit for repair and, despite its undisputed advantages, there are also disadvantages among them, for they cause more morbidity, and represent a limited resource; thus motivating many health care specialties to try and find sound bone replacement, that add to the advantages, reducing these disadvantages^18^. There are in the market commercially consolidated substances, still under test, that have been used as effective bone replacements, with some expected properties, such as: biocompatibility, osteoconduction, osteoinduction^5^.

Among bone replacements deriving from bone tissue, we chose to use human demineralized bone in this research project, since it is one of the most used in dentistry, because it is homogenous^19^, and one of the few in this category that is available for commercial use [5][5][1]. Moreover, it presents good results which are scientifically proven^13^,^20^. This debatable property of the human-origin demineralized bone, of promoting osteoinduction is one more important piece of data to be used in this research^13^.

Another category of bone replacement options is made up of materials which do not come from bone tissue, such as ceramics (hydroxyapatite, tricalcium-phosphate), bioactive glass, calcium sulphate, polymers, amongst others. These biomaterials are synthetic materials developed in order to stimulate and promote bone repair, avoiding the use of grafts, or even minimizing its use^9^. In this category we stress the biopolymers for presenting, in recent years, a tremendous scientific development^20^. Among these we have the castor oil derived polyurethane [6][6][2] that was used in this research for having its development totally done in Brazil, of vegetal origin and for these reasons, it is of low cost9. Also, for presenting scientific proof of biocompatibility and osteoinduction potential^21^, ^22^, ^23^.

In order to test these bone replacements we chose a prospective, comparative and randomized animal experimental model. This option was due to the fact that this was the most adequate model for experimentation with bone replacements, before clinical assays^9^,^24^.

Results have also shown that even at the end of the longest slaughter period, 15 weeks after cavity preparation, spontaneous bone regeneration was enough to promote complete bone repair in the borders of the control cavity. This does not corroborate with the results from other authors when the cavity receives no specific treatment, except for irrigation and filling up with blood from the animal itself^14^,^23^. Such fact is somewhat curious; having seen that the cavity created had size compatible with a critical defect. This type of defect is so called because it does not allow for spontaneous cavity repair^24^. Based on this fact, we may suggest that, maybe be, this size considered critical, is not really true. Thus, we need additional studies that may consistently determine the size of a critical defect.

During the four week interval, initial period of assessment for our work, it was possible to identify the presence of particles of this material surrounded by fibrous connective tissue inside the surgical bone cavity, both in the castor oil polymer group and in the demineralized bone group, meaning little time for complete bone repair. In this initial observation period, the first tissue formed was the connective fibrous tissue, filling up the bone defect space created; natural characteristic of this regeneration phase^24^, there was still osteoid and osteoblastic paving in small quantities. Similar results are described for the castor oil polymer^22^ and for human origin demineralized bone^3^,^24^.

Within seven weeks, in both groups, castor oil polymer and demineralized bone, we noticed particles of material involved by fibrous connective tissue and bone. Here we noticed a bone neoformation from the defect margins; however, still with a large amount of connective fibrous tissue in the demineralized bone group; while in the polymer group there was almost complete bone defect repair by mature bone tissue around the implanted material, and such fact was also observed by Garcia (2000)^24^ in regards of demineralized bone, and by Ueda et al. (1996)^22^, assessing the castor oil polymer. A better result was seen in the polymer group when compared to the demineralized bone.

After fifteen weeks, an almost complete bone repair was observed in all the groups, with border union in the experimental groups and bone union in the control group by thinning of the neoformed bone; and this thinning was not seen only in the polymer group. When there was defect repair in the control group, the body was unable to keep the same bone thickness formerly present in the region, despite having completely repaired it. In the same study, we could observe an important resorption of demineralized bone particles, showing that because of this it was also not possible to keep the same “pre-defect” thickness” of the cranial vault, differently from the polymer group which presented itself as a material with almost no resorption at this time, thus being incorporated to the bone defect in a biocompatible way, keeping greater repair thickness, since it became an integral part of it, even making us believe it is a material that may be used in repairing bone defects in which the bone contours must be preserved.

In a general way, we noticed, through histology, that the material tested had a positive influence on bone neoformation in the defects. In both experimental groups, osteoconduction was the pathway responsible for regeneration. This bone neoformation happened, mainly through the borders, from the periphery of the pre-existing bone towards the center, different from multi ossification sites, which is a characteristic that happens during the osteoinduction process. The demineralized bone presented similar behavior to the one found in other studies^3^, ^24^. These results were different from the ones found by Mulliken et al. (1981)^25^, in which 100% of the cavities filled by demineralized bone matrix were completely filled by neoformed bone tissue after two weeks, Nonetheless, surgical bone cavities in these two experiments were made in the cranial vaults of rats and had 4mm of diameter, enough size, having seen the goal of determining the ideal bone defect for the bone replacement study of Hollinger and Schimitz (1987)^23^ who stressed the need to use a bone defect of enough size so as not to have spontaneous regeneration, thus according to this protocol, research has to investigate defects of at least 8mm of diameter.

Carvalho et al. (1997)^12^ and Ueda et al. (1996)^22^ found, in their studies with castor oil polymers, results very similar to the ones attained in this investigation, bone neoformation by osteoconduction. As to the resorption, in our study, castor oil polymer was very little resorbed at the end of fifteen weeks. Ignácio et al. (1997)^9^, after producing bone defects in the radius of 34 rabbits, and replace them by castor oil polymer rods, observed the almost complete polymer replacement by bone, where in the 16 week group, both bone ends (distal and proximal) were practically fused to the central region. Now, Carvalho et al. (1997)^12^ and Ueda et al. (1996)^22^ did not see total or almost total resorption in their studies. Nonetheless, Ueda et al. (1996)^22^ used rabbit tibias for bone defects and evaluated bone repair after only 40 days. Carvalho et al. (1997)^12^ made the last analysis within 6 weeks of the initial procedure. All observation periods were less than the ones we used.

Finally, in bone regeneration, the use of osteoconduction bone replacements is not advisable in the repair of large defects. Duguy et al. (2000)^26^ justified this based on bone growth, in this type of material, being limited to the implant borders. Because of this, Lewandrowsky et al. (1999)^27^,^28^ already suggested the combination of an osteoconduction matrix with osteoinduction material, such as growth factors in order to obtain complete bone repair.

Besides, studies similar to this one must be carried out using additional radiographic, histomorphometric and immunohistochemical evaluations in order to provide the necessary tools to allow for qualitative and quantitative comparisons among the different materials investigated.

## CONCLUSION

Within the goals of this study, and based on the results attained, we may conclude that:

1-Histologically, both the control and experimental groups (I and II) presented an increase in bone neoformation along time, which was faster in the control groups when compared to the experimental ones.

2-Both materials tested were biocompatible, the human demineralized bone matrix was resorbed much faster than the castor oil polymer, which seems to be of slower resorption, as observed in the histologic analysis carried out in the 4, 7 and 15 week periods.
